# Decision fatigue experience of front-line nurses in the context of public health emergency: an interpretative phenomenological analysis

**DOI:** 10.1186/s12912-024-02163-w

**Published:** 2024-08-13

**Authors:** Shan-Shan Dong, Kun Wang, Ke-Qiang Zhang, Xing-Hui Wang, Jian-Hang Wang, Subinur Turdi, Jia-yu Yang, Li He, Rong Yan, Yue-Wei Li

**Affiliations:** 1 Hepatopancreatobiliary Surgery Department, General External Center, First Hospital of Jilin University, Changchun, Jilin Province China; 2 Pediatric Respiratory Department, Children’s Hospital, First Hospital of Jilin University, Changchun, Jilin Province China; 3 Medical Department, Second Hospital of Jilin University, Changchun, Jilin Province China; 4 Department of Nursing, First Hospital of Hebei Medical University, Shijiazhuang, Hebei Province China; 5 Operating Room, Second Hospital of Jilin University, Changchun, Jilin Province China; 6 School of Nursing, Jilin University, No. 965 Xinjiang Street, Chaoyang District, Changchun, Jilin Province 130021 China

**Keywords:** Public health emergency, Nurse, Decision fatigue, Phenomenology

## Abstract

**Background:**

Decision fatigue is a new concept in the field of psychology and refers to a state of fatigue alongside impaired cognitive processing and emotional regulation ability. Previous studies have confirmed that nurses are prone to decision fatigue, and nurses who experience decision fatigue may implement nursing measures that are inconsistent with clinical evidence, thus affecting patients’ benefits. COVID-19, as a large-scale global public health emergency, increased the workload and burden of nurses and aggravated decision fatigue. However, the factors leading to decision fatigue among nurses have not yet been identified.

**Methods:**

This study is guided by interpretative phenomenology. During the epidemic period of COVID-19: From November 2022 to February 2023, a one-to-one, semi-structured in-depth interview was conducted among nurses with decision fatigue experience who were participating in front-line work in Jilin Province using homogenous sampling. The interview recordings and related data were transcribed into text within 24 h, and data analysis was assisted by NVivo 12.0 software.

**Results:**

After a total of 14 front-line nurses were analyzed in this study, The thematic level reaches saturation, the findings present a persuasive and coherent narrative, and the study is terminated, and finally extracted and formed three core themes: “Cognition, influence and attitude of decision fatigue”, “Approaching factors of decision fatigue” and “Avoidant factors of decision fatigue”.

**Conclusion:**

This study confirmed that decision fatigue was widespread in the work of front-line nurses, affecting the physical and psychological health of nurses, the quality of nursing work, the degree of benefit of patients and the clinical outcome. However, nursing staff do not know enough about decision fatigue, so the popularization and research of decision fatigue should be strengthened. Improve the attention of medical institutions, nursing managers and nursing staff.Some suggestions are put forward for the intervention of decision fatigue through personnel, task, tool and technology, organization and environment.

**Supplementary Information:**

The online version contains supplementary material available at 10.1186/s12912-024-02163-w.

## Background

The term “Disease X” was first coined by the World Health Organization in 2018, and “Disease X” is not a disease, but a label that refers to the potential for a hypothetical, as yet unknown pathogen to cause a severe international pandemic. “X disease” has the characteristics of “high lethal, fast infection, easy mutation”, and there is great uncertainty in its occurrence and development. that millions of people had died after the outbreak of COVID-19. The social, economic and political shock is still reverberating. The COVID-19 is the first “X disease”,outbreaks of new pathogens and epidemics are only a matter of time, not whether they will occur.At the end of 2019, the WHO declared COVID-19, first discovered in Wuhan, China, as an international public health emergency.The rapid spread of COVID-19 has led to great challenges for both medical institutions and health care workers. In order to deal with the epidemic, the nursing work environment is rapidly adjusted: nurses are providing nursing services for patients with the strange disease, the proportion of personnel is unbalanced, the supply of personal protective equipment is limited, the difficulty of nursing is greatly increased, and weaken the emotional regulation ability of nursing staff [1, 2].

As a clinical nurse, the researcher has participated in the front-line care of the epidemic.In the special working environment of the fight against the epidemic, researchers and other front-line nursing staff fighting against the epidemic have repeatedly experienced difficult decision-making or taking conservative decisions, insufficient physical endurance, weakened attention control and emotional regulation ability, anxiety and other conditions.According to the literature, this phenomenon belongs to the category of decision-making fatigue, and We learned that the complex working environment and psychological burden can easily lead to decision-making fatigue. Decision-making fatigue may have adverse effects on the psychological state of nurses, clinical decision-making and even the prognosis of patients [3–8]. In order to improve the mental health status of nursing staff and provide safer care for patients, decision-making fatigue deserves in-depth research and exploration.

Decision fatigue is an emerging concept in the field of psychology, which refers to the impaired ability of decision-making and control behavior caused by repeated decision-making behavior. It is a fatigue state [3] with impaired cognitive processing and emotional regulation ability. It is found that bad mood and cognitive burden are all important factors that cause or aggravate decision-making fatigue [9]. Individuals who experience decision fatigue usually show a tendency to make impulsive or conservative decisions after weakening the ability to weigh the pros and cons, making the decision quality decline [10, 11]. Several studies have shown that decision fatigue is common among caregivers at work [12, 13].

This study focuses on the decision-making fatigue experience of front-line nurses in the context of public health emergencies (COVID-19), aiming to explore the cognition and attitude of decision-making fatigue and the feelings and influence of decision-making fatigue. It provides a basis for formulating coping strategies in the face of public health emergencies improve the quality of clinical nursing and empower clinical nursing management.

## Method

### Design

Interpretive phenomenological analysis (IPA) [14] guided the design and development of this study.Interpretive phenomenology was founded by J. A. Smith on the basis of phenomenology and other theories. Phenomenology, hermeneutics and specific orientation are the three important ideological sources of interpretive phenomenology [15, 16].To explore the cognition, attitude, influence, feeling and factors of decision fatigue from the perspective of front-line nurses.This study was carried out under the guidance of the Qualitative Research Reporting Standard (SRQR) [17] and the IPA quality evaluation standard proposed by Smith to ensure the quality and rigor of the research [16].

### Setting and sample

The particular approach of interpretative phenomenology requires that studies focus on smaller, more homogeneous samples [14]. This study adopts homogenous samples to select a group of cases with relatively similar internal components for research, aiming to conduct in-depth discussion and analysis of cases with similar experience in the research phenomenon, so as to obtain the experience of homogenous samples. The main recruitment channels for respondents were multiple wechat groups of “treatment and nursing medical teams”, and a small number of subjects were recruited by phone, mail and face to face. The interviewees were recruited and studied from November 2022 to February 2023. We selected research subjects in the same region and during the same period of the epidemic of the new coronavirus. The subjects were trained according to the same version of the “Epidemic Prevention and Control Guidelines” of Jilin Province and carried out anti-epidemic work. The research team believed that the internal components of the research subjects were similar.Interpretive phenomenology holds that data collection and analysis can stop when the findings can be presented as a persuasive and coherent narrative [18]. In this study, after interviewing and analyzing the data of the 14th front-line nurse, the research team agreed that the extracted content had reached the purpose of this study and could be presented as a persuasive and coherent narrative, and the data collection and analysis was stopped.

The inclusion criteria were as follows: ① Registered nurses working in clinical practice;②Nurses who were participating in the front-line clinical work of COVID-19 in the designated treatment unit at the time of their participation in this study;③ Nurses with decision fatigue were identified after scoring with Decision Fatigue Scale (Chinese version) ; ④Voluntarily signing of an informed consent and voluntary participation in this study; ⑤Nurses who could afford to spend 1–2 h of continuous time to be interviewed.

The exclusion criteria were as follows: ① Nurses who were not in direct contact with the COVID-19, such as nursing managers and other support (logistics) positions.

### Data collection

According to the Chinese version of Decision Fatigue Scale [19] and relevant literature, the preliminary interview guidelines outline was formulated in combination with the research purpose and the opinions of experts in this field. Before the formal interview, two interviewees were selected according to the inclusion and exclusion criteria, and pre-interviews were conducted for these two front-line nurses. Two pre-interviews ensured clarity of questions and robustness of interview design. The data from the pre-interview are not included in the thematic analysis, but are helpful to ① revise the content of the interview outline; ② Ensure the interviewees’ comfort level with sensitive topics; ③ Confirm that the interview time of 45–90 min is reasonable [20].According to the pre-interviews results, the research team met face-to-face to discuss and revise the interview guidelines outline to determine the final interview guidelines outline. Before the formal interview, first explain the purpose, content and format of the interview to the interviewee. Ask the front-line nurses whether they know the concept of decision fatigue, explain the definition of decision fatigue to the interviewees according to their understanding, and list the actual cases of decision fatigue. To achieve a clear definition of decision fatigue. The interviewees were asked about their wishes, signed the informed consent after obtaining the informed consent, and filled in the general information questionnaire for the interviewees. Number the interviewees according to the order of interview, conceal their real names, and privacy of the interviewees.The interviewee has the right to withdraw or refuse to answer questions at any time during the interview. Relevant information about the environment or interviewees’ tone, pauses, etc. observed in the field records are recorded in the form of field notes. An interview ends when both the researcher and the interviewee confirm that nothing new needs to be mentioned. Each interviewee was interviewed only once. Each interview lasted 45–90 min, and all interviews were conducted by the same investigator with no third person present during the interview.

Because the COVID-19 can spread through the respiratory tract. Therefore, out of a total of 14 interviewees, only two interviewees were interviewed face-to-face, respectively in the office area of the designated treatment unit (enclosed space without a third person present) and the apartment of the front-line nurses (enclosed space without a third person present). Face-to-face interviews can more directly observe the body language of the interviewees. By using the video call function of wechat to complete the interview with five interviewees, we can also observe the body language of the interviewees. Meanwhile, our research finds that it is easier to capture the inner world of the interviewees when using the video call function of wechat to complete the interview than face-to-face interview. Seven interviewees were interviewed by telephone. Although they could not observe their body language, they could also record their voice, intonation, mood fluctuations and other expressions.

In the process of inviting research subjects, Two front-line nurses declined to participate in the study. One front-line nurse did not specify the reason for the refusal, and the other refused because of concerns about the leakage of personal information.A total of 14 front-line nurses were interviewed, each of whom was interviewed once, and no repeated interviews were conducted.

### Data analysis

The data were analysed using IPA. IPA is an ideographic approach that seeks to obtain an understanding of the lived experiences of individuals by exploring their unique meaning-making and the meanings they ascribe to particular phenomena. Smith argues that, as humans, we have always been engaged in interpretative meaning-making. Interpretation is the basic structure of our intentional lives, and is therefore not only acceptable, but inevitable. Since the IPA sees the primary role of the researcher as an invitation to the participant to share his construction of meaning, to witness its expression, and thus to understand it, this aligns the IPA with Heidegger’s hermeneutic phenomenology [15, 16, 21].

In this study, IPA was used to obtain an in-depth understanding of the experiences with decision fatigue reported by front-line nurses working in clinical response efforts in the context of COVID-19. The six-step IPA method introduced by Smith was used for data analysis. The specific steps of this analytical method are as follows:

Step 1: The interview transcripts are read repeatedly. The recordings of the interview are listened to repeatedly. Then, the interviews are transcribed and sent to the interviewed nurses to verify their accuracy. Throughout research process, the researchers record their observations and ideas at any time in the form of field notes and memos.

Step 2: Preliminary annotation and analysis. Initial coding was conducted independently by the investigator himself and under the guidance of the supervisor (research team leader). The discussion process is not designed to convince each other, but to make a comprehensive and thorough analysis of the nature of the phenomenon, to ensure that the final decision made can explore the nature of the phenomenon at a deeper level.

Step 3: Generate themes, pooling similar codes into subthemes, and the research team meets to discuss the subthemes.

Step 4: Determine the correlation between the subthemes, form the theme, and the research team meets to discuss and determine the theme.

Step 5: Proceed to the next case analysis. After a new interview, repeat the first four analysis steps to develop the previous hypercoordinated theme by extracting new data or a new theme.

Step 6: Search for searched in crossover cases, and the research team met to discuss the analysis results until all topics were endorsed to ensure accurate and comprehensive analysis results. However, this step is not the last step in the data analysis process, and the analysis continues even when the results are recorded.

### Rigor

The data was supplemented and repeatedly confirmed by the interview recording, field notes and memos to maximize the data. In the process of interpretation and analysis of data, the circular principle of interpretative phenomenology is applied, and self-reflection and criticism are constantly carried out in order to achieve the deepest level of interpretation.

Research team members have deep learning qualitative research, and the research team leader has rich experience in qualitative research. The study process and results were carefully reviewed by an expert in the field of qualitative research who was not a member of the study team and followed that expert’s recommendations. The coding was done independently by the investigator himself, and the research team met in regular meetings to ensure that the analysis achieved the study objectives.

This study was reported in accordance with the Qualitative Research Reporting Standard (SRQR) checklist. Meanwhile, Smith developed quality evaluation guidelines for IPA research in 2011 [16]. It is pointed out that qualified IPA research should meet the following four criteria:①Clearly indicate that the research follows the three theoretical principles of IPA (phenomenology, hermeneutics and specific orientation); ② The research process is clear and transparent, including how to select research objects, how to conduct interviews and analysis processes; ③ Clear and reasonable; ④ There is sufficient evidence to support each topic. This study follows the above four criteria to ensure the rigor of this study.

### Ethical considerations

This study was approved by the Ethics Committee (2,022,110,308). In this study, all methods were performed in accordance with the Declaration of Helsinki. All participants signed informed consent forms and volunteered to participate in the study, and they could withdraw from the study at any time. All participants were promised that their information would be kept confidential, and no participants dropped out of the study. To ensure the confidentiality of the participants’ information, the participants were coded according to the order of the interviews, with a numerical code identifying participant categories and the order in which they were interviewed. Access to the recordings and other related materials was restricted to members of the research team stored only on the first author’s computer in the form of encrypted files.

## Results

Fourteen nurses who ranged in age from 22 to 41 years old and who had working experience ranging from 2 to 23 years were interviewed. The 14 participants were all from northeast China, and the sample included 11 women and 3 men drawn from 5 different work units, as shown in Table 1. After reading the transcribed text repeatedly, the research team extracted a total of 3 themes and 10 subthemes, as shown in Table 2.

**Table 1:** Characteristics of the fourteen nurses

ID	Interview channel	Gender	Age	Years of working	Profession title	Position	Education degree	Marital status	Next generation information	Class of hospital	Decision fatigue score
N1	face to face	Female	30	5	nurse	Clinical nurse	Master	Single	none	Class III, Level A	13
N2	Wechat	Female	34	11	supervisor nurse	Clinical nurse	Bachelor	Married	Her son is a pupil	Class III, Level A	15
N3	phone	Female	36	9	supervisor nurse	Clinical nurse	Master	Married	Her son is a kindergartner	Class III, Level A	16
N4	Wechat	Male	28	5	nurse	Clinical nurse	Bachelor	Married	His son is a kindergartner	Class III, Level A	18
N5	Wechat	Female	35	10	nurse	Clinical nurse	Junior college	Married	Her son is a baby	Class III, Level A	13
N6	WeChat	Female	33	10	nurse	Clinical nurse	Junior college	Married	Both of her daughters are pupils	Class III, Level A	18
N7	phone	Male	22	2	nurse	Clinical nurse	Bachelor	Single	none	Class III, Level A	18
N8	phone	Female	33	6	nurse	Clinical nurse	Junior college	Single	none	Class III, Level A	16
N9	phone	Female	33	10	nurse	Clinical nurse	Bachelor	Married	Her son is a kindergartner	Class III, Level A	13
N10	WeChat	Female	32	7	supervisor nurse	Clinical nurse	Bachelor	Single	none	Class III, Level A	15
N11	phone	Female	41	23	supervisor nurse	Clinical nurse	Junior college	Married	Her son is a pupil	Class III, Level A	18
N12	phone	Male	25	3	nurse	Clinical nurse	Junior college	Single	none	Class III, Level A	13
N13	phone	Female	32	10	nurse	Clinical nurse	Bachelor	Married	Her son is a baby	Class III, Level A	13
N14	face to face	Female	32	9	supervisor nurse	Clinical nurse	Bachelor	Married	Her son is a kindergartner	Class III, Level A	15

**Table 2:** Themes and subthemes

Themes	Subthemes			Code number	No. of participants
Theme 1: Cognition, influence and attitude of decision fatigue	Cognition of decision fatigue	Total ignorance or vague understanding		14	14
	It has no negative effect on individuals and shows a neutral attitude	It is inevitable and has no negative effect		3	3
	It has a negative impact on individuals and falls into psychological difficulties	Worry, suffering, confusion, regret, guilt, self-blame, unfulfilled self-worth		32	14
Theme 2: Approaching factors of decision fatigue	Self depletion and negative energy of others	Imprisoned, exhausted, overdrawn self depletion	Emotional exhaustion	18	12
			The solidified image	1	1
			Physical exhaustion	9	8
			Mental elasticity	1	1
		Estranged, apathetic, whining others are negative	Complex team	2	2
			Communication barriers	4	4
			Negative energy	6	6
	High-load, high-difficulty task	Overload work		16	9
		High-difficulty work		12	8
	A caged bird lacking experience or tools	Lack of experience or tools		14	12
		Caged bird - Protective gear is both armor and shackles		9	8
	An imperfect system	The relevant system is not yet perfect		8	8
		Temporary positions are not ownership, no sense of belonging		4	4
	Internal environment constraint	Work environment factors		8	8
Theme 3: Avoidant factors of decision fatigue	External environment control: macro-policy control	From the government’s macro policy regulation		7	7
	External environment control: care of medical institutions	Care from the medical establishment		5	5

### Cognition, influence and attitude of decision fatigue

#### Cognition of decision fatigue

Through the analysis of interview data, it was found that the front-line nurses had different cognition degrees of decision fatigue. Some interviewees said they were completely unaware of the concept of decision fatigue. *“Never heard of it.” (N1) “Is it fatigue?” (N10)* At the same time, some interviewees have a vague understanding of the concept of decision fatigue.

“Does decision fatigue mean making decisions when you’re tired?” (N5) Some interviewees believe that decision fatigue is real. “There must be, too many of them.” (N2) “Yes, I think we should all have that”. (N11)

It is found that most interviewees do not know or understand the concept of decision fatigue through the interview, which is related to the fact that decision fatigue is in its initial development stage in China and has not yet attracted the attention of medical institutions and nursing managers, and the concept has not yet been popularized. On the other hand, nursing staff have been working in high-load and high-pressure environment with the concept of execution and obedience for a long time, and the problem has not been paid attention to because of the imprisonment of image. At the same time, it was again verified that decision fatigue was widespread among front-line nurses, and the incidence and severity of decision fatigue gradually increased over time. It should be paid attention to by medical institutions, nursing managers and front-line nurses.

#### It has no negative effect on individuals and shows a neutral attitude

Sometimes I will be assigned to finish the disinfection of the place where the protective clothing has been removed, This is a very heavy workload and I need to wear two layers of protective clothing to complete the work.At the beginning, I was very serious, and every corner was carefully disinfected, but later I felt a little bit faint, as if I could not hold on, I felt a quick wipe.We are asked to pressure wipe disinfection, to the end, feel particularly hot, are suffocating, may not pay attention to the pressure, wipe quickly. The main thing is there’s no study that says pressurization works. (N1)

In the case of external conditions that do not allow high-quality completion of all work, many interviewees said that the more decisions they make in the front-line work of the epidemic, the more conservative they tend to be, and decision fatigue is inevitable. However, the decision fatigue of front-line nurses in the work that they considered to be relatively unimportant or unnecessary did not have a significant negative impact on the clients, so they took a neutral attitude. During the interview, I deeply realized the helplessness of front-line nurses in the fight against the epidemic. The overloaded work under the special working environment increased the incidence of nurses’ decision-making fatigue.

#### It has a negative impact on individuals and falls into psychological difficulties

Negative psychology is common among front-line nurses in the fight against the epidemic. During the interview, it was found that the vast majority of nurses would fall into certain psychological difficulties after decision-making fatigue.

Causing adverse consequences, involving the liability of the hospital, and it is their own reason to cause such a situation, it is really not right. (N2)

Interviewees mentioned that the adverse consequences caused by decision fatigue will be very worried about being held accountable, and the worried mood will last for a period of time, which is easy to increase the psychological burden.

Some elderly people are difficult to communicate, always press the pager, I have been to many times, and ask me the same question over and over again. Later, she pressed the pager again, and I was busy, thinking that she had nothing to do, so I didn’t go. And then I thought about her all the time. Don’t be miserable. (N10)

After experiencing decision fatigue due to the need to make repeated decisions, interviewee was worried about whether patients had care needs due to conservative decision making and not reaching out to them. In the follow-up work and life, the nurse felt very tortured and exhausted, and it was difficult to concentrate on the next work.

A patient intravenous infusion of drugs ended, press the caller, was too busy, I did not go back to the computer to check the doctor’s order, later found that the patient has a bottle of medicine, and gave the patient a needle, I feel particularly embarrassed, but the patient is fine, always said nothing. (N9)

The interviewee mentioned that the occurrence of decision fatigue increased the patient’s unnecessary pain and brought her psychological distress. The word “particularly” represents her regret, and the word “always” reflects the nurse’s tolerance and understanding of the patient, which makes her feel more guilty and increases her psychological burden.

Each patient has many kinds of oral medicine, that day was too busy, did not check the number of pills remaining, the results of the next shift colleagues found that the number of drugs is not enough, and asked me? It really bothers me. (N3)

The interviewee mentioned that some work was not completed in high quality after decision fatigue, and the word “bother” reflected nurses’ remorse and regret. In the process of shift transition, the burden of the nurses in the next shift was increased, and the negative emotions and attitudes shown by the nurses in the next shift also caused the respondents to blame themselves and fall into psychological difficulties.

The widespread existence of decision fatigue causes front-line nurses to fall into multiple psychological dilemmas such as worry, suffering, confusion, regret, guilt, self-blame, and weakened sense of self-worth, which increases psychological burden, reduces work enthusiasm, and weakens sense of self-worth. Timely intervention is needed to improve the physical health of nursing staff, avoid the occurrence of psychology-related diseases, improve the quality of nursing, and avoid the adverse outcomes of patients.

## The approaching factors of decision fatigue

### Self-depletion and the negative energy of others

#### Emotional exhaustion

All the patients were calling me, the busier and messier, accidentally spilled the iodine, my nursing records were all wet, I was really stupid, more collapsed, you know? Collapse to the extreme, already want to cry, but also have to work, only myself, I can only complete the most critical work, more delicate care I can only decide to give up, I have no time to make a decision, that time is really make people want to cry. Going to work is like fighting. (N14)

The nursing notes were soaked in iodine, the interviewee used *“collapse to the extreme”* to describe the mood at that time, even when the interview recalled that experience, her eyes still lit up with tears, her voice was trembling, and her expression was helpless. *“Wanting to cry without tears”* indicates the interviewee’s inner frustration and helplessness. *“I have to work*,* just myself…… Work is like a war”*, emptying my body, going all out to fight a person, no teammates, can not retreat, isolated, it seems that a “strong me” must rely on constant consumption of their own to defeat another “weak me.” The interviewee stressed that she did not have time to make decisions, indicating the onset of decision fatigue. In the front-line work against the epidemic, attention should be paid to the psychological status of nursing staff, timely relief of anxiety, tension, frustration, helplessness and other negative emotions, so as to avoid the occurrence of decision-making fatigue.

#### The solidified image

I don’t eat when I’m hungry at noon, I don’t go to the toilet, and I waste a protective suit when I eat. At the end of the day, my legs go weak, my heart is pounding and I don’t want to make a decision. (N9)

Front-line nurses generally have a strong sense of responsibility. In order to avoid increasing the burden on colleagues, they work against the clock and increase their physical consumption. Clinical nurses have been confined to the image of *“thrifty*,* tough guy*,* I have to persist*,* I can do”* and so on, constantly self-reinforcing, not timely self-adjustment, after four hours of continuous work appeared *“legs weak*,* heart pounding”* symptoms, serious physical loss leads to do not want to make decisions, decision-making fatigue. It is suggested that work in a special environment wearing protective clothing, and four-hour shift is a reasonable scheduling mode.

#### Physical exhaustion

There is relatively stuffy, no windows, no doors, very stuffy, but we are strictly wearing N95 masks, with a face screen, wearing next door clothes.I was very stuffy, very hot, I was already breathing difficulty, with the elderly patients over and over again to say, explain, over and over again ink, hearing patients need to communicate loudly, my state is very poor. The long night shift and this suit, the personnel is relatively dense, so it is difficult. In order to protect myself from infection, I dare not drink water, and it is impossible for me to take off my mask. Talking to patients, I will present a dry mouth, brain hypoxia state, I do not want to say, can say as little as possible. (N10)

The interviewee said that the working environment in the designated treatment units for the COVID-19 is very closed and personal protection is very strict. With the extension of working hours, nurses are in a state of sweltering, lack of water and oxygen for a long time, and thus their physical strength and endurance decline. For elderly patients facing communication difficulties in this state, the constant repetition of nursing education content will accelerate the energy consumption of nurses. From the tone of the interviewed nurse, we can feel her helplessness, the interviewee said that if the physical condition allows, she would like to provide more detailed care for patients. Public health emergencies make the entire medical and health system face great challenges. In the face of similar disease prevention and control, places with good ventilation and appropriate temperature and humidity should be set as designated places for treatment as far as possible. If conditions permit, elderly patients should be approved to be accompanied by family members or equipped with nurses in order to carry out life care and reduce unnecessary physical and emotional consumption of front-line nurses against the epidemic. In addition, attention should be paid to the frequency and time of night shift, so that nutrition supply, adequate sleep and abundant physical strength of front-line nurses can effectively alleviate the occurrence of decision fatigue.

#### Mental elasticity

Decision fatigue I think everyone has it, I think it is related to personal characteristics, usual work habits, if you don’t adjust yourself, decision fatigue will definitely happen. (N5)

Decision fatigue generally exists in nurses on the front-line of the epidemic response, but the degree of performance is not the same in different people, which is related to personal characteristics and work habits. The interviewee emphasized that positive self-regulation can reduce the incidence of decision fatigue, and individual self-regulation in negative environments is related to mental resilience, and mental resilience is positively related to adaptability. Therefore, medical institutions can regularly organize stress simulation training to improve nurses’ ability to regulate the external environment and improve their ability to cope with negative events such as pressure, frustration and trauma.

#### Complex team

The temporary establishment of the comprehensive wing, the various hospitals together, the usual work habits are not the same, the number of nurses to participate in the anti-epidemic experience is also different. Team coordination is poor, there are more contradictions, it is too easy to make decisions fatigue, physical and psychological fatigue. (N4)

The interviewee mentioned that nursing teams formed by temporary staff from different medical institutions can accelerate the onset of decision fatigue due to different working habits and standards, different nursing experience, poor teamwork, poor communication, and apathy from colleagues. Therefore, the temporary support team should be divided into different levels and regions according to the nurses’ previous work experience and post competency, and do a good job of pre-job business training and psychological counseling.

#### Communication barriers

Elderly patients are difficult to communicate, hard of hearing, speaking in dialects that I can’t understand, which is a big factor.There is also such a patient is particularly weak, or no teeth and so on, so that he speaks I can not understand, I can only guess what he wants to do. I was really busy and had no time, so I had to give up. (N6)

The interviewee mentioned that elderly patients generally have problems such as hearing loss and poor expression, and there are great obstacles in communication with elderly patients, and the difficulty of nursing is greatly increased, and nurses need to spend more time to achieve effective communication, which will inevitably increase the workload of nursing staff and accelerate the occurrence of decision fatigue. It is suggested that elderly patients can increase the degree of smooth communication by increasing the accompanying family members, routinely wearing dentures, hearing-aid, etc., when necessary, but also improve the psychological security and medical experience of elderly patients.

#### Negative energy

Let the patient wear a mask, the environment is noisy, the normal volume is not clear, I want to say loudly, do not understand, I have to explain to him, or do not listen to me. I told him clearly that the patient next to me was positive and asked him to wear a mask, but he didn’t understand, so I turned around and left, and he didn’t wear a mask in the end, but I decided not to tell him, I tried my best and I told him. (N14)

In the treatment of the COVID-19, the treatment area is divided according to the severity of the disease. In the mild treatment area, the number of patients is relatively high and the environment is noisy, so nurses need to raise their voice to nurse the patients. In the mild treatment area, patients have insufficient understanding of the disease and poor cooperation, and adopt the attitude of disapproval, disregard and non-implementation, which seriously affects the enthusiasm and professional identity of nurses. At the same time, the environment is noisy, and the front-line nurses need to consume more physical energy to explain the work. Repeated explanations cause physical energy consumption, emotional regulation ability is impaired, The result is decision fatigue. Unified disease-related education work can be carried out through mobile devices or electronic screens in medical institutions to enhance the safety awareness of patients and their families, reduce the negative energy transmission of front-line nursing staff, increase the cooperation of patients and their families, reduce the degree of self-loss of nursing staff, and delay the occurrence of decision fatigue.

### High-load, high-difficulty task

#### Overload work

Severe illness has a special area called the disability zone, every time to eat, because they can not eat independently, need to feed, each person is responsible for more than 10 disabled patients, there are many other work, and need to feed, they eat slowly, but also choke, eat not satisfied, and negative emotions.Sure decision fatigue! This is the most difficult time to get through, the anxiety, the desire to get the job done, this is the most painful. (N2)

The interviewee mentioned that she cared for more than 10 severely disabled elderly people at the same time, caught up the time to be fed, and the incidence of decision fatigue increased significantly. Elderly patients eat slowly, which needs to occupy a longer working time of nurses, resulting in passive reduction of other work time. Elderly patients are prone to coughing when eating, and have the risk of lung infection and even suffocation, which increases the difficulty and psychological burden of nursing for front-line nurses. In addition, The interviewee mentioned that elderly patients is prone to a lot of negative emotions, which is bound to have a negative impact on the heart of nurses. The interviewee recalled that experience with obvious complaints, sadness, helplessness, grievance negative emotions, but there is no way to vent. It suggests that in the front-line of anti-epidemic, we should arrange the ratio of beds to nurses reasonably, and if conditions permit, we should equip nurses to assist nursing work. Pay timely attention to the psychological state of nurses, regularly assess the workload of front-line nurses, pay attention to the workload of front-line nurses, and avoid the occurrence of decision fatigue caused by high workload.

#### High-difficulty work

Pushing the patient from that position to the door, the process is a mountain, because there are particularly many beds in the middle, the whole process is very tedious. The space is particularly small, it is difficult to pass, and many items need to be found while using, which is particularly inconvenient. I’m already so busy, I’m so devastated, I can’t cry, I have to keep working, and going to work is like fighting a war. (N14)

During the outbreak of the COVID-19, the volume of patients increased dramatically. The interviewee mentioned that it would be particularly difficult if patients needed to move their beds outside for check-ups. The interviewee described the work as *“climbing mountains”*, reflecting that the work is time-consuming and laborious, the work is extremely difficult, and the self-depletion is aggravated. In addition, the inadequate preparation of nursing materials also increases the physical consumption of nurses, wastes limited working time, and inevitably leads to the shortening of other nursing operations. *“Collapse”*, *“want to cry without tears”*, *“have to continue to work”*, *“work and war like”*, let us not help but think of the image of nurses over the years: *“tough woman”*, nurses seem to always be in the front of the one, such psychological suggestion in the early work can effectively alleviate the occurrence of decision-making fatigue. However, working under high load and high pressure for a long time will inevitably lead to impaired emotional regulation ability and lead to decision fatigue. It suggests that we should sort out and simplify the front-line work flow of anti-epidemic regularly, timely supplement nursing materials, pay attention to the psychological state of nurses, and do a good job of psychological counseling.

### A caged bird lacking experience and tools

#### Lack of experience or tools

At the beginning of the work is not very familiar, leading to companions are waiting for me, I feel this is particularly bad. I hope not to drag my partner’s legs, work together, we can also go out together, rather than everyone waiting for me. It was a time of intense anxiety and stress, and it was difficult to make decisions. (N5)

The high infectivity of COVID-19 requires caregivers to wear protective clothing to work, and the team should remove protective clothing and leave the infected area at the same time in designated places.Interviewee mentioned that they could not finish their work within the specified time due to lack of experience when they first started to work on the front-line of the epidemic, resulting in the situation that colleagues waited for the interviewee to leave work together. This makes the interviewee feel anxious, and over time, the psychological burden gradually increases, eventually leading to the occurrence of decision fatigue. It suggests that we should pay attention to pre-job training and experience exchange and sharing, and nursing managers can also take past experience as an important factor in personnel scheduling.

#### Caged bird - protective gear is both armor and shackles

Wearing protective clothing adds to my sense of security, equivalent to the armor that stops bullets. It’s just hard to get dressed, and you don’t eat, you don’t drink. At first, it is OK, but after a long time, the body will be uncomfortable, which will affect the quality of care. (N2)

The interviewee mentioned that *“protective clothing is like armor”*, indicating that although wearing protective clothing can reduce the risk of infection and increase the sense of security of front-line nurses, it also brings a lot of inconvenience and discomfort to front-line nurses. The reason may be that the steps of wearing and taking off protective clothing are tedious and time-consuming. Nurses do not want to waste their working time by changing protective clothing and do not want to add extra work pressure to colleagues because of their departure. Therefore, they would rather endure physical discomfort such as thirst, hunger and discomfort than change new protective clothing. Some interviewees also mentioned that because protective clothing is more expensive, they do not want to waste public resources and refuse to replace it, so there is a situation in which they force themselves to complete their work by wearing protective clothing, and there is a risk of affecting the quality of care.

### An imperfect system

#### The relevant system is not yet perfect

There is no uniform standard for disinfection, this person says it should be disinfected this way, that person says it should be disinfected that way. I don’t know which one makes sense, so it’s more prone to decision fatigue. (N1)

The interviewee mentioned that she was responsible for the environmental disinfection of the treatment area when she was working in the anti-epidemic front line, and the disinfection process did not specify the disinfection details. Two experienced colleagues worked with her before and after, but the disinfection methods of the two people were different, and the significance of doing so was not explained to her. The interviewee mentioned that decision fatigue was more likely to occur in the environmental disinfection in the following work. It can be seen that when nurses clearly know the actual requirements and significance of a certain job, they can improve their cognitive processing ability and effectively alleviate the occurrence of decision fatigue. The work content of the anti-epidemic front line is relatively unfamiliar to nurses, so they should do a good job of pre-job training, and explain the key points and significance of a specific job while describing the work process and content is crucial. The imperfect system increases the difficulty of the implementation of nursing work, speeds up its own loss, and finally leads to the occurrence of decision fatigue.

#### Temporary positions are not ownership, no sense of belonging

Most people think they are here to help, not to work as hard and seriously as they do in their own department, and I think it has something to do with it. (N3) Of course, I want to go back to my own department, but I feel like I’m not here after all, I don’t even have a cabinet, I only have a small box when I change clothes, and I don’t have a sense of belonging. I think that’s a big reason for conservative decisions. (N12)

Many nurses mentioned that *“we are just here to help”*, saying that it is good to finish the work, and should not ask for too much. Under the guidance of this philosophy, repeated decisions tend to be more conservative. Chinese traditional thought is deeply rooted, thousands of years of historical and cultural heritage let us pay more attention to the concept of “home”, the sense of belonging can stimulate the fighting spirit of nurses, nursing managers should consider how to increase the magnetism of nursing places to nurses, in order to increase the sense of belonging of nurses.

#### Internal environment constraint

When I first went there, the environment gave me a lot of pressure, there were too many people, too many patients, the ground is full of patients, there is a lot of pressure to work, definitely decision-making fatigue. (N10)

The interviewee mentioned that the COVID-19 was raging, the number of patients increased sharply, *“the ground is full of patients”*, and the poor working environment caused her a lot of work pressure, resulting in decision-making fatigue. The working environment will have an impact on the mental and physical of employees. The depressed office environment, the lack of air circulation, and the pressure of work will cause mental damage to employees to a certain extent. A comfortable working environment, such as the right temperature, humidity, lighting, noise levels, and a clean and organized office space, helps employees stay focused and happy, which increases productivity. Medical institutions can work out emergency plans for public health emergencies in advance to avoid such situations.

### Avoidant factors of decision fatigue

#### External environment control: macro-policy control

A number of interviewees mentioned that the policy support of the government and medical institutions was the driving force for them to complete the front-line work against the epidemic.

Participating in front-line nursing work can be promoted to a supervisor one year in advance, otherwise I dare not think of promotion. Live alone everywhere, participate in front-line nursing work and subsidies, very good. (N9)

The interviewee mentioned that according to the promotion regulations of their units, the nurse did not fully meet the conditions for promotion to the next level, but the government issued a policy of *“Care for front-line employees”*, according to which medical institutions optimize the promotion channel and provide special support for front-line nurses against the epidemic, so that they can obtain promotion opportunities after successfully completing the anti-epidemic work. The interviewee used the phrase *“I dare not think?”* It reflects that the promotion conditions of nursing workers are higher and more difficult, and the introduction of this policy has given her great encouragement, greatly improving the work enthusiasm, and the improvement of work enthusiasm has largely delayed the occurrence of decision fatigue. In addition, the interviewee mentioned, *“Live alone every where, participate in front-line nursing work and subsidies, very good.” (N9)* The nurse was single and lived alone, with relatively few family ties. Many interviewees reported that in the front-line work of the anti-epidemic response, with the extension of support time, the concern for family members would aggravate negative emotions such as anxiety and suffering, and aggravate the occurrence of decision-making fatigue at work. Therefore, macro-policy control can bring positive incentives to front-line medical workers and effectively improve their work enthusiasm. In addition, it is also necessary to consider the implementation of family-oriented policy support to reduce the psychological burden of front-line personnel.

#### External environment control: care of medical institutions

Seeing the public’s praise for us on the mobile phone makes me feel an instant energy. Also, I had diarrhea two days ago, reported to the team leader, did not expect the big leader actually called me to care about me, and gave me a bunch of medicine, I was particularly moved. (N10)

External praise and affirmation can improve the professional identity of front-line nurses, increase their sense of self-worth, and greatly improve their work enthusiasm. The love, encouragement, understanding and support from leaders and colleagues enhance team cohesion, increase work happiness, and effectively relieve the pressure brought by negative psychology.

My son is in primary school, usually homework is my management, when I came to think about what to do, I did not expect the hospital actually arranged online tutoring classes, too touched. (N5)

The interviewee mentioned that she usually focuses on her children in addition to work, and once worried about her children’s academic problems because she participated in the anti-epidemic work, she could not devote herself to work. The medical institution offered to set up online tutoring classes for the children of front-line nurses in the anti-epidemic work, which to some extent solved the nurse’s worries.

## Discussion

The rapid spread of COVID-19 has led to an unprecedented global public health crisis, and medical institutions and medical workers are facing great challenges. This study deeply explored the cognition, attitude, feeling and influence of decision fatigue among frontline nurses in the context of public health emergencies, and confirmed once again that decision fatigue is widespread among front-line nurses in the response to the epidemic, effectively affecting the physiological and psychological status of nurses, the quality of nursing work, and even the outcome of patients. Therefore, decision fatigue deserves the attention of medical institutions, nursing managers and front-line nurses. Appropriate strategies can be formulated according to the practical experience of decision fatigue of front-line nurses, and intervention can be carried out from the aspects of personnel, tasks, tools and technology, organization and environment.

During the period of COVID-19, a large number of studies have shown that the psychological pressure of front-line nurses against the epidemic is greater, and the anxiety score is significantly increased. However, our study found that interviews, on the one hand, improved participants’ cognition of the concept of decision fatigue, and on the other hand, alleviated the anxiety of front-line nurses.

During COVID-19, our research found that preferential promotion, financial subsidies and family support from medical institutions or superior leaders can effectively improve the work enthusiasm of front-line nursing staff and alleviate decision fatigue.

### Cognition, influence and attitude of decision fatigue

Decision fatigue is still in the initial stage of development in China. In the course of this study, it was found that although front-line nurses did not understand the concept of decision fatigue, after the explanation of the researchers, nurses all said that decision fatigue was widespread in front-line nursing work against the epidemic, This is consistent with the findings of Ann et al. [22].The occurrence of decision fatigue is closely related to the special background of public health emergencies. In the future, we should strengthen the popularization of the concept of decision fatigue, enhance the cognition degree of nursing staff to decision fatigue, identify the signs of decision fatigue as soon as possible, deeply understand the impact of decision fatigue, and correct improper behavior in time. At the same time, we should strengthen the research on decision fatigue, broaden the research field, and explore the effective measures to avoid decision fatigue in many aspects and levels, so as to reduce the influence of decision fatigue to the greatest extent.

Interviewees showed different attitudes towards decision fatigue. Decision fatigue did not have a negative effect on some nurses, and the nurses maintained a neutral attitude towards it. Front-line nurses are more likely to suffer from decision fatigue in tasks that they think are relatively unimportant or do not directly affect the clinical outcome of patients. It is necessary to strengthen the training and interpretation of the basis or significance of the relevant work, which can avoid the occurrence of decision fatigue to a certain extent.

A number of interviewees also said that decision fatigue would have a certain negative impact on them after the occurrence of decision fatigue, leading to psychological distress of the respondents, weakening the sense of self-worth, increasing the psychological burden, affecting the quality of care, and even affecting the clinical outcome of patients. Therefore, they believe that decision fatigue deserves early intervention and intervention. Nursing staff should make timely self-adjustment to relieve multiple pressures, and nursing managers should also pay timely attention to the psychological status of front-line nurses, increase in-depth communication, detect problems in time, and intervene as soon as possible.

### Approaching factors of decision fatigue

This study found that self depletion and others negative energy; High-load, high-difficulty tasks; A caged bird lacking experience or tools; An imperfect system; The internal environment constraint are the approaching factor of decision fatigue.

Personnel: emotional regulation, physical endurance, mental resilience, interpersonal interaction, and negative energy transmission can accelerate the occurrence of decision fatigue among front-line nurses, This is consistent with the findings of Hatami et al. [23, 24].The support time is too long, the risk of self-infection is increased, and the nursing needs of patients are multiple aggravating emotional exhaustion; Self-reinforcing trapped in a captive image; The tightness of protective equipment, the alternation of cold and hot ambient temperature, and the inability to supply energy at any time lead to physical exhaustion; Continuous self-regulation to build mental resilience; Temporary establishment of support team, different departments and different experience personnel combination; Older people, mood swings and other difficult to communicate; The propagation of negative emotions and energy both accelerate the nurses’ self-depletion and eventually lead to the occurrence of decision fatigue. Decision fatigue generally exists in the work of clinical nurses, which can lead to the tendency of passive, avoidant and impulsive decision making. Ego depletion is associated with the depletion of self-control resources triggered by emotional regulation and cognitive function. The occurrence of decision fatigue will affect nurses’ reasoning and decision-making ability, leading to the reduction of decision-making quality. Nursing staff need to maintain a certain concentration to complete the observation work, and the cognitive impact caused by decision fatigue should be paid attention to. Therefore, attention should be paid to the psychological state of front-line nurses in clinical response to the epidemic, and phased training and psychological counseling should be done to ensure timely and effective communication between nursing managers and front-line nurses. Nurses should also timely adjust their psychological and physiological states and pay attention to the positive effects of adequate sleep, abundant physical strength, interval rest and glucose supplement. In the future, appropriate measures should be taken to reduce the self-depletion of front-line nurses against the epidemic, and team training should be emphasized to prevent the spread of negative emotions caused by the negative energy of others and thus the occurrence of decision fatigue.

Tasks: Large patient flow, limited staffing, lack of supervision, supporting staff or inadequate infrastructure will increase the tasks of front-line healthcare workers. The amount of multiple tasks will lead to multiple decisions, which will damage the quality of decisions, increase the work pressure, and further aggravate the occurrence of decision fatigue. Overload workload and difficult work are important factors that cause decision fatigue. The ratio of nurse to patient should be appropriate, and the position setting of nursing staff should consider multiple factors such as post competence, work load and work difficulty. At the same time, the front-line work of anti-epidemic needs to maintain sufficient physical strength. Many nurses mentioned that four hours is the limit of continuous working time wearing protective equipment, and the psychological and physiological state will be seriously decreased after four hours. The longer a nurse works continuously, the more decisions they make and the more conservative their decisions tend to be, and strategically scheduling (frequent, short) breaks is the best way to ensure that decisions remain efficient throughout the shift.

Tools and technology: nurses lack experience in front-line nursing work against the epidemic, and young nurses are not skilled in nursing operation skills and lack emotional regulation ability, which makes them more prone to decision-making fatigue. On the other hand, the lack of materials such as turning pads and information equipment has increased the amount of tasks and the difficulty of work. In addition, the protective clothing is airtight, which brings great discomfort to the front-line nursing staff, increases physical exertion, and accelerates the occurrence of decision fatigue. Therefore, it is necessary to regularly organize experience sharing meetings for front-line nursing staff to enrich front-line work experience, and pay attention to personnel matching based on work experience and post competence. Ensure the supply of tools for front-line work against the epidemic, and strengthen the applicability of nursing tools. Attention should be paid to the wearing standards of protective equipment to ensure the comfort of front-line personnel to the greatest extent.

Organizational aspects: overload workload, tasks that do not match nurses’ nursing experience and post competence, and work processes that are not adjusted in time aggravate the occurrence of decision fatigue. Medical institutions and nursing managers should timely adjust the work flow with the deepening of the understanding of the disease, and at the same time should do a good job of interpretation when issuing the relevant work flow and system, to ensure that front-line nurses are clear about the basis and significance of the formulation of a certain job, and avoid unnecessary work to increase the burden of nurses. According to the nurses’ work experience and post competence, the management should be strengthened, the tasks should be allocated reasonably, and the staff should be matched reasonably. At the same time, in principle, dual-income medical and health workers with elderly people and children will not be arranged to work on the front-line of the epidemic.

Environmental aspects: A poor work environment can make decision makers vulnerable to additional stress, which weakens their decision-making ability and leads to decision fatigue. Public health emergencies are often accompanied by rapid changes in the environment, so we should pay attention to the appropriate temperature and humidity, and try to avoid the loss of front-line nurses brought by the environment to fight the epidemic. In addition, pay attention to the nutrition supply and physical supplement of front-line nursing staff, adequate sleep and appropriate glucose supplement can effectively alleviate the occurrence of decision fatigue.

### Avoidant factors of decision fatigue

This study found that external environment regulation (macro-policy regulation and care from medical institutions) is the circumvention factor of decision fatigue. Many interviewees said that the government and medical institutions’ policy support and care for front-line nursing staff and families have solved the worries of front-line nurses, reduced the psychological burden, improved the enthusiasm of work, and also avoided the occurrence of decision fatigue to a certain extent. Pay attention to the humanistic care of front-line nurses to reduce the psychological burden. We can appropriately increase the performance tilt support, priority employment, exception application, and title tilt policies. After the end of the front-line work, people can take concentrated rest, rest at home and paid leave in accordance with the requirements of epidemic prevention and control.

### Suggestion and enlightenment

The outbreak and global epidemic of COVID-19, the first “X disease”, have made all regions of the world face a situation of strained medical resources, shortage of medical personnel and difficult nursing environment. Nurses are generally faced with heavy physical and psychological burdens, and the incidence of decision-making fatigue has increased significantly. This study identified the approaching and avoiding factors of decision fatigue, and provided a basis for formulating corresponding strategies for other public health events such as “X disease” that may occur in the future.

Nursing managers and front-line nursing staff should pay attention to the problem of decision fatigue and understand the potential harm of decision fatigue. On the premise of not affecting the safety of patients, psychological intervention measures such as interrupting continuous working hours, taking a short rest, carrying out relaxation training and mindfulness therapy can alleviate the occurrence of decision fatigue.

Setting up the energy supply area and appropriately placing the food or drink supplied with sugar can alleviate the occurrence of decision fatigue by inhibiting physical exertion.

The purpose of reducing decision fatigue is to improve work efficiency by means of information technology. Decision support system and electronic nursing records can effectively improve decision fatigue by avoiding decision errors caused by repeated decisions.Actively shape and enhance the nurse’s personal sense of accomplishment, while ensuring a certain benefit package to cope with a variety of emergency medical decisions and changing work environment.

### Limitations

This study is a qualitative study, the participants are a single country, a single region, a single culture sample, the research results may have some regional differences. The COVID-19 lasted for three years, and front-line nurses had certain changes in their knowledge of the virus and their work mentality. The interview time of this study was in the middle and late period of the COVID-19, which ensured the homogeneity of samples to a certain extent, but the research results could not cover the overall period of COVID-19. In addition, the nuances within the group of participants suggest that each participant is following his or her own spiritual path, which makes thematic analysis somewhat challenging.

## Conclusions

This study conducted a qualitative study on the decision fatigue experience of front-line nurses in the context of public health emergency (COVID-19). This paper analyzes and summarizes the cognition, influence, attitude approaching factors and avoidance factors of decision fatigue. It fills a gap in relevant research fields and provides a basis for developing response strategies to public health emergencies such as “Disease X”.

This study confirmed that decision fatigue is widespread in front-line nurses’ work against the epidemic, and affects the physical and psychological health of nurses, the quality of nursing work, the degree of benefit of patients and the clinical outcome. However, nursing staff do not know enough about decision fatigue, so the popularization and research of decision fatigue should be strengthened, and medical institutions, nursing managers and nursing staff should pay more attention to it.

By exploring the cognition, attitude, feeling and influence of front-line nurses on decision fatigue, this study analyzed the approaching and avoiding factors of decision fatigue. Some suggestions are put forward for the intervention of decision fatigue through personnel, task, tool and technology, organization and environment.At the same time, we suggest that: ① Nursing managers and front-line nursing staff should face up to and pay attention to the problem of decision fatigue and understand the potential harm of decision fatigue. On the premise of not affecting the safety of patients, psychological intervention measures such as interrupting continuous working hours, taking a short rest, carrying out relaxation training and mindfulness therapy can alleviate the occurrence of decision fatigue. ②Setting the energy supply area and appropriately placing the food or drink supplied by sugar can alleviate the occurrence of decision fatigue by inhibiting physical exertion. ③ Improve work efficiency by means of information technology to achieve the purpose of reducing decision fatigue. Decision support system and electronic nursing records can effectively improve decision fatigue by avoiding decision errors caused by repeated decisions.④ Actively shape and enhance the personal sense of accomplishment of nurses, while ensuring certain welfare benefits to cope with a variety of emergency medical decisions and the changing work environment.

It is hoped that the results of this study can be combined with quantitative research in the future to verify the effectiveness of intervention measures and effectively alleviate the degree of decision fatigue of front-line nurses.

### Electronic supplementary material

Below is the link to the electronic supplementary material.

Supplementary Material 1

Supplementary Material 2

## Data Availability

Data sets in qualitative interviews are confidential and will not be shared.
