# Supplementary material for: Decision fatigue experience of front-line nurses in the context of public health emergency: an interpretative phenomenological analysis
Source: BMC Nurs. 2024 Aug 13;23:553. doi: 10.1186/s12912-024-02163-w (PMC11321180; doi:10.1186/s12912-024-02163-w)
Supplement: Supplementary file 1 — Supplementary Material 1 [file 12912_2024_2163_MOESM1_ESM.doc]

Interview guidelines outline

Introduction

Hello, I am a graduate student in the School of Nursing, Jilin University, and my research topic is the decision fatigue experience of frontline nurses in the context of public health emergencies. Objective of this study:To explore the cognition and attitude of frontline nurses to decision fatigue and their feelings and influence after decision fatigue.Research methods:Using homogenous sampling, selecting nurses with decision fatigue experience to conduct one-to-half structured interviews to collect data, sign informed consent, and obtain consent from recording.

Respondents' cognition of decision fatigue

1. Are you too tired or stressed to make a decision? Talk about it specifically?

2. Do you ever find it difficult to make a decision because you can't concentrate? Talk about it specifically?

3. Do you have a hard time understanding information and making decisions based on it? Talk about it specifically?

4. Are there times when you don't have enough confidence in yourself to make the right decision? Talk about it specifically?

5. Have you ever had to put a lot of effort into making a decision? Talk about it specifically?

6. Do you ever feel like someone else should make decisions for you? Talk about it specifically?

7. Have you ever been unable to decide which option is best? Talk about it specifically?

8. Have you ever made a decision without careful consideration? Talk about it specifically?

9. Do you have a hard time making decisions because you are in a bad mood? Talk about it specifically?

10. How do you understand decision fatigue?

Respondents experience decision fatigue

11. Can you share your specific experience of decision fatigue in your frontline work?

12. Can you explain the scene in detail?

13. What do you think caused decision fatigue at that time?

14. How do you feel when you experience decision fatigue?

The impact of decision fatigue on respondents

15. Do you feel that decision fatigue has any impact on you?

Respondents' attitude towards decision fatigue

16. Do you think decision fatigue can be prevented or mitigated? What do you think can be done?

17. Do you think decision fatigue deserves attention? Why?

18. How do you now understand decision fatigue from our conversation?

19. Is there anything else you would like to share with me about decision fatigue?
